# Opportunistic screening data for early prediction of GDM in Northern Chinese women: a multicenter machine learning study

**DOI:** 10.1038/s41598-026-42700-y

**Published:** 2026-03-09

**Authors:** Haotian Zhai, Lifan Che, Tao Xu, Ning Li, Chuansheng Li, Jie Xin, Xinru Zhang, Yao Liu, Yongqi Li, Zhe Ma, Yang Li

**Affiliations:** 1 Zichuan District Hospital, Zibo City, Shandong Province China; 2 Yidu Central Hospital of Weifang, Qingzhou City, Shandong Province China; 3 Department of Medical Ultrasound, The First Affiliated Hospital of Shandong First Medical University & Shandong Provincial Qianfoshan Hospital, Shandong Medicine and Health Key Laboratory of Abdominal Medical Imaging, 16766 Jingshi Road, Lixia District, Jinan City, Shandong Province China; 4 Wendeng District Maternal and Child Health Care Hospital of Weihai City, Weihai City, Shandong Province China; 5 The London School of Economics and Political Science, London, UK

**Keywords:** Opportunistic screening, Gestational diabetes mellitus, Visceral adipose tissue, Early prediction, Machine learning, Endocrine system and metabolic diseases, Endocrinology, Predictive markers, Ultrasonography

## Abstract

To develop a machine learning model for early prediction of gestational diabetes mellitus (GDM) using routinely available first-trimester clinical and ultrasound data in Northern Chinese women. This multicenter prospective cohort study enrolled pregnant women from three hospitals in Northern China. We integrated first-trimester maternal characteristics and standardized ultrasound measurements of subcutaneous (SAT) and visceral (VAT) adipose tissue thickness. After addressing potential selection bias via inverse probability weighting, we employed a genetic algorithm (GA) for robust feature selection and evaluated five machine learning classifiers (XGBoost, ANN, SVM, MLR, RF). Model performance was assessed on an internal test set and an independent external validation set, with AUC as the primary metric. The GA consistently selected BMI, SAT, and VAT as core predictive features. The model combining GA-selected features with XGBoost demonstrated the highest performance, achieving an AUC of 0.962 on the internal test set and 0.878 on the external validation set, with corresponding sensitivities of 0.90 and 0.70, and specificities of 0.942 and 0.935, respectively. It significantly outperformed models using other feature selection methods or classifiers (all *P* < 0.001). The model exhibited robust stability across various sensitivity analyses. A machine learning model based on readily accessible first-trimester indicators provides an effective tool for early, opportunistic GDM risk stratification in Northern Chinese women.

**Supplementary Information:**

The online version contains supplementary material available at 10.1038/s41598-026-42700-y.

## Introduction

Gestational diabetes mellitus (GDM), defined as hyperglycemia first detected in pregnancy that does not meet the diagnostic criteria for overt diabetes, is a common complication with significant implications for maternal and offspring health^1–6^. Current international guidelines recommend diagnosis via a 75-g oral glucose tolerance test (OGTT) at 24–28 weeks of gestation. However, this late diagnosis limits the window for early interventions aimed at mitigating associated complications, such as fetal overgrowth and hypertensive disorders. Identifying high-risk women in the first trimester is therefore critical for enabling timely, preventive strategies.

Existing research on early GDM prediction has primarily focused on clinical risk factors or second-trimester biomarkers^7,8^. Recently, abdominal fat distribution, measured via ultrasound, has emerged as a promising early indicator of metabolic dysfunction. Maternal visceral adipose tissue (VAT) and subcutaneous adipose tissue (SAT) thickness are linked to insulin resistance and GDM pathogenesis^9–12^. However, the integration of these routinely acquired, first-trimester ultrasound measures with standard clinical data into a predictive model—particularly within a resource-conscious, opportunistic screening framework—remains underexplored. Such an approach leverages existing prenatal care infrastructure without imposing additional testing burden, making it highly applicable to diverse clinical settings, including those with limited resources.

This study developed an early-pregnancy GDM prediction model by integrating routinely collected first-trimester clinical and ultrasound data. Our approach utilized opportunistic screening—leveraging existing prenatal care infrastructure—which was considered particularly relevant for resource-limited settings where specialized testing might be unavailable. The model aimed to identify high-risk pregnancies during the first trimester, enabling a shift of the intervention window from post-diagnosis management to early prevention. This earlier timeframe allowed for the implementation of feasible preventive strategies, such as basic dietary guidance and physical activity counseling, which could help mitigate subsequent pregnancy complications.

## Methods

### Subjects

This study was a multi-center prospective cohort investigation.This multicenter prospective cohort study was conducted in Northern China, enrolling pregnant women from three hospitals in Shandong Province, China, representing distinct regional healthcare settings: Zichuan District Hospital of Zibo City (a district-level hospital), Yidu Central Hospital of Weifang in Qingzhou City (a county-level hospital), and Shandong Provincial Qianfoshan Hospital in Jinan (a provincial tertiary hospital). Pregnant women who underwent prenatal examinations and had delivery records in medical institutions across various regions and levels from June 2023 to June 2024 were selected as research subjects. The inclusion criteria were as follows: singleton pregnancy, gestational age between 8 and 14 weeks, absence of severe internal or surgical complications, and the ability to comply with the entire prenatal care process. The exclusion criteria included women under 18 years of age, multiple pregnancies, pregnancies complicated by gestational hypertension, fetal or chromosomal abnormalities, hyperthyroidism or hypothyroidism, and pre-existing diabetes.

The GDM group was determined based on the World Health Organization (WHO)-recommended 75 g OGTT standards during 24 to 28 weeks of gestation, specifically, fasting blood glucose ≥ 5.1 mmol/L, 1-hour post-glucose-load blood glucose ≥ 10.0 mmol/L, and 2-hour post-glucose-load blood glucose ≥ 8.5 mmol/L. A diagnosis of GDM was made if any of these blood glucose values exceeded the established thresholds. The control group consisted of pregnant women with normal OGTT results during the same timeframe. It should be noted that in our clinical setting, universal first-trimester glucose screening for the detection of overt diabetes is not routinely performed. Therefore, women with undiagnosed pre-existing diabetes may not be identified until the standard 24–28 week OGTT. In such cases, if their glucose levels meet the WHO criteria for GDM but not for overt diabetes, they are managed as GDM.This study was submitted to the ethics committee and received an approval number. All methods were carried out in accordance with relevant guidelines and regulations, including the Declaration of Helsinki. Written informed consent was obtained prior to participation.

### Data collection

During the sampling and inclusion process, including fasting blood glucose levels, were routinely documented in outpatient prenatal records. Clinical risk factors potentially associated with GDM, such as age, body mass index (BMI), family history of diabetes, smoking history, parity, and previous pregnancy complications (e.g., miscarriage, stillbirth, macrosomia, premature birth, eclampsia, pre-eclampsia), were also recorded. These data were meticulously detailed when pregnant women established their prenatal care records. The accuracy of the information was ensured by inquiring about medical history and reviewing prior physical examination reports. Family history of diabetes was obtained through careful questioning of the pregnant women and their relatives (parents, siblings, etc.) regarding their diabetes status. In cases where the pregnant woman was uncertain about her family history, efforts were made to contact relatives for verification or to consult family medical records.

### Standardized ultrasound measurement protocol

Certified sonographers performed all abdominal fat measurements using a rigorously standardized protocol. A high-resolution linear array probe was employed to obtain precise measurements at the umbilical level during the end-expiratory phase to ensure consistency. The probe was positioned perpendicular to the skin surface with minimal applied pressure to avoid tissue compression, while maintaining optimal contact for accurate imaging.

**Fig. 1:**
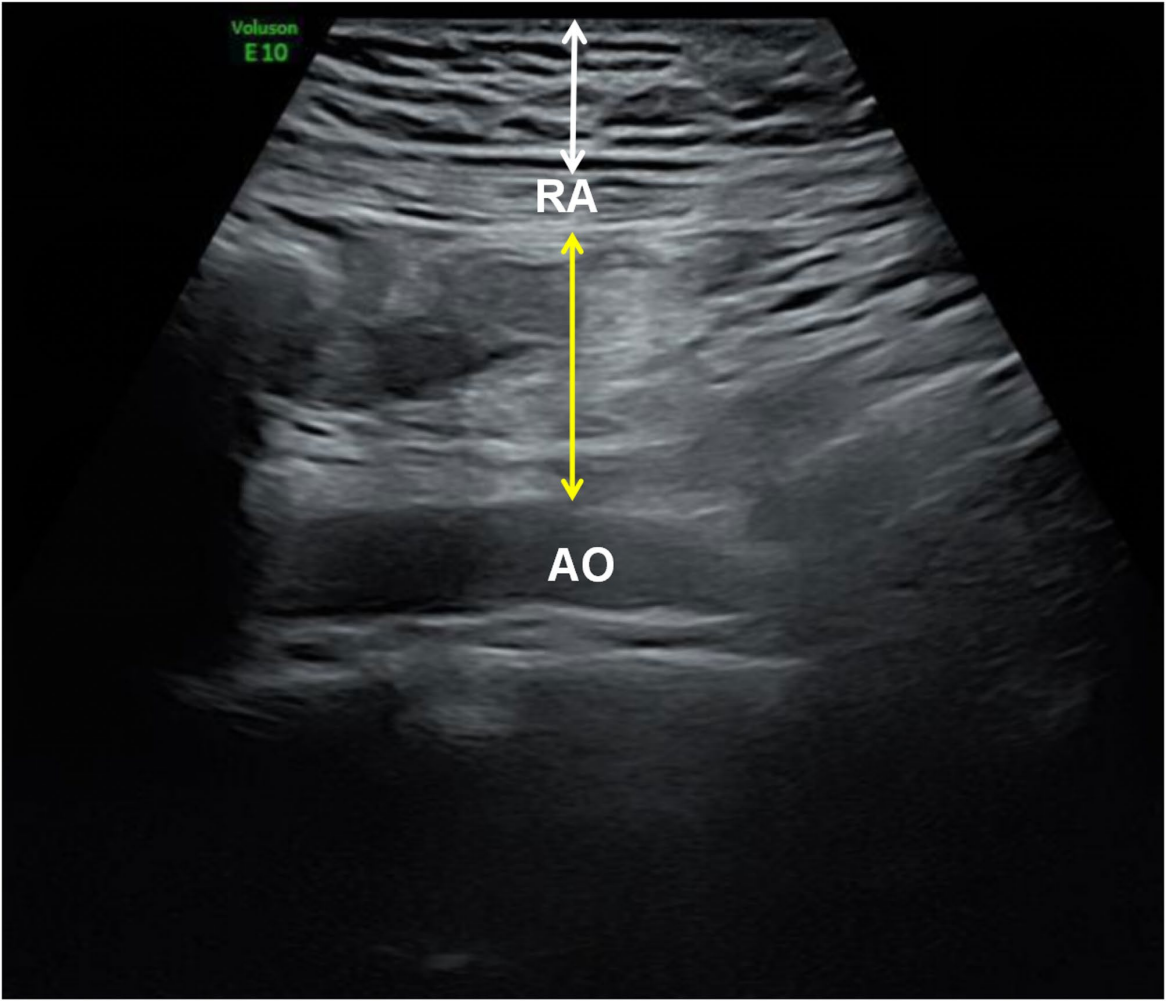
Ultrasound measurement of maternal abdominal SAT(white arrow) and VAT (yellow arrow) indicators. RA, rectus abdominis muscle; AO, abdominal aorta; SAT, subcutaneous adipose tissue; VAT, visceral adipose tissue.

For subcutaneous adipose tissue thickness measurement, we recorded the distance from the dermal layer of the skin to the outer edge of the rectus abdominis muscle. Visceral adipose tissue thickness was determined by measuring from the inner edge of the rectus abdominis muscle to the anterior wall of the abdominal aorta (Fig. 1). At each measurement site (umbilical level or maximal abdominal fat location), three consecutive measurements were obtained by the same operator. These measurements were then averaged to derive the final values for both SAT and VAT, with strict quality control requiring < 5% intra-observer variability to ensure measurement reliability. This standardized approach minimized potential errors and enhanced the reproducibility of our fat thickness assessments.

### Data partitioning and preprocessing

The modeling data were derived from the integrated dataset of Zichuan District Hospital and Yidu Central Hospital. They were divided into a training set (60%) and an internal test set (40%) using stratified random sampling. Data from Qianfoshan Hospital was separated from the cohort as an external validation set. Continuous variables (age, gestational weeks, BMI, SAT, VAT) were standardized by Z-score method, and categorical variables were factorized.

### Bias correction based on inverse probability weighting (IPW)

Given that the incidence of GDM in our cohort was significantly higher than the theoretical incidence in the general population, potential selection bias existed. Therefore, we performed correction using Inverse Probability Weighting. First, propensity scores were calculated using Lasso logistic regression, and the common support between cases and controls was examined via density plots. To prevent model instability caused by extreme weights, the weights were winsorized (truncated at the 5th and 95th percentiles) and normalized. These weights were applied to all subsequent feature selection and model training.

### Feature selection strategy

Genetic Algorithm (GA) was used for feature selection, with the area under the receiver operating characteristic curve (AUC) of the IPW-weighted XGBoost model serving as the fitness function. To ensure robustness and avoid local optima, five independent GA runs were conducted with different mutation and crossover rate combinations. The final features included in the model were the intersection of the results from the five runs. For comparison, Recursive Feature Elimination (RFE) and stepwise regression were also employed. RFE used the same IPW-weighted XGBoost setup, while stepwise selection used IPW-weighted logistic regression with AIC as the criterion.All feature selection procedures were accomplished on training set to avoid potential overfitting risks.

### Machine learning model development

Based on the selected features, five machine learning models were developed: Extreme Gradient Boosting (XGBoost), Multivariate Logistic Regression (MLR), Weighted Support Vector Machine (WSVM), Random Forest (RF), and Artificial Neural Network (ANN). All models incorporated the sample weights derived from IPW during training to mitigate the impact of selection bias on prediction probabilities.

### Model evaluation and statistical analysis

Model performance was evaluated on the internal test set and the external validation set, with the evaluation metrics including AUC, Accuracy, Balanced Accuracy, Sensitivity, Specificity, F1 score, and Macro F1 score. The optimal thresholds were determined by maximizing Youden’s index (Youden’s index = sensitivity + specificity – 1). Pairwise comparisons of model performance were conducted using the one-sided Bonferroni corrected DeLong test, with the GA-feature-based XGBoost model as the benchmark. Descriptive statistical analyses and hypothesis tests were performed with SPSS 26.0. Machine-learning workflows were implemented in R using scikit-learn, XGBoos.

## Results

### Basic characteristics of the study subjects

**Fig. 2:**
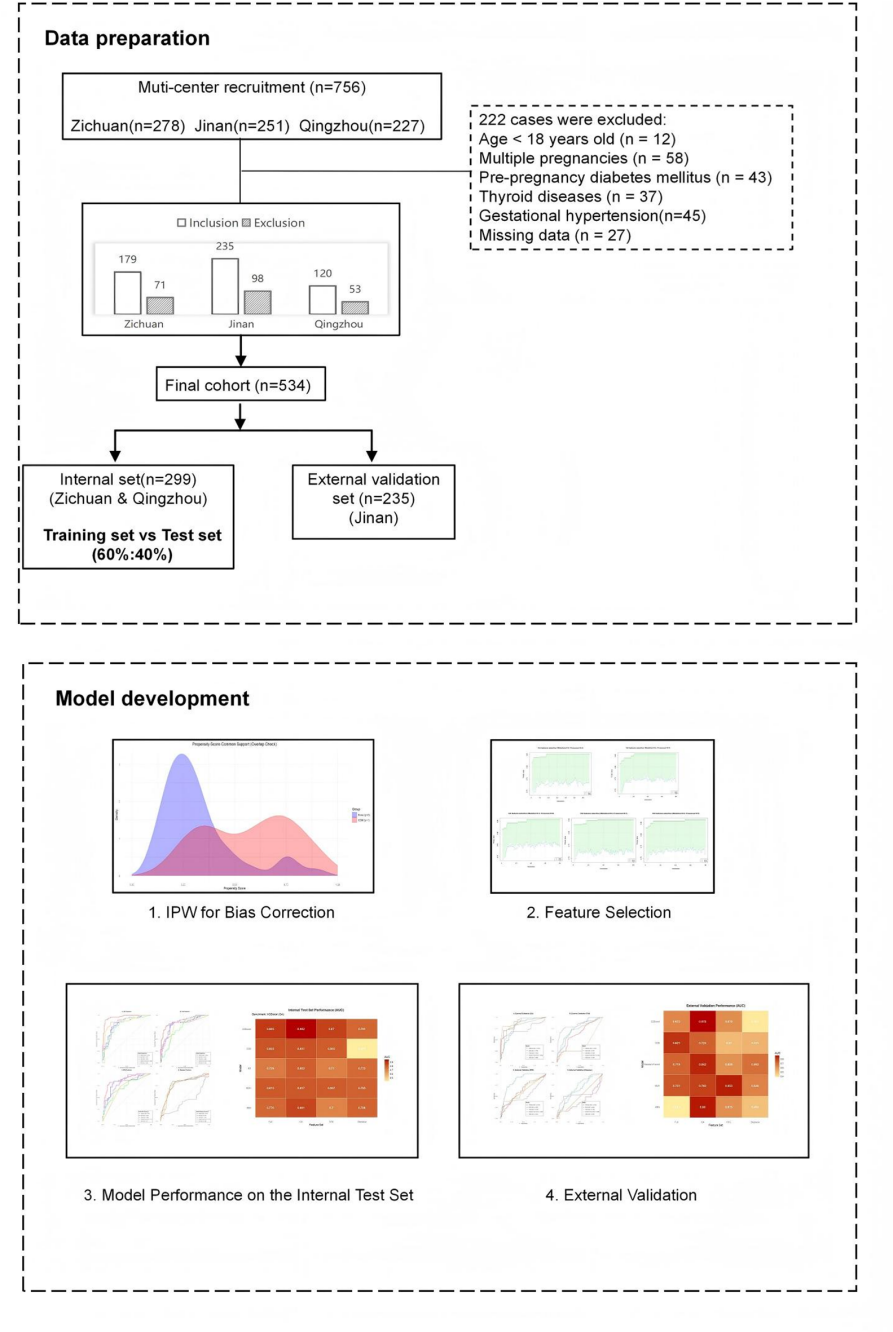
Flowchart of the study design. The diagram illustrates the number of women assessed for eligibility, excluded (with reasons: pre-pregnancy diabetes, multiple gestation, age < 18 years, thyroid disease, gestational hypertension, other exclusions, loss to follow-up, or incomplete data), and finally included from each of the three participating centers.

The flowchart of the study design presented the detailed process of model development (Fig. 2). A total of 222 participants were excluded due to predefined criteria: age < 18 years (*n* = 12), multiple pregnancies (*n* = 58), pre-pregnancy diabetes (*n* = 43), thyroid diseases (*n* = 37), gestational hypertension (*n* = 45), and missing data (*n* = 27). Finally, 534 pregnant women were included in this study. Among them, 177 cases were in the case group (pregnant women with GDM), and 357 cases were in the control group (pregnant women without GDM). The distribution of the basic characteristics of the pregnant women in the two groups was shown in Table 1.

**Table 1:** Comparison of the basic characteristics of the study subjects.

General information	Normal group (*n* = 357)	GDM group (*n* = 177)	*P*
Age (years)	28.42 ± 4.84	29.14 ± 4.66	0.880
Gestational weeks (weeks)	10.62 ± 1.71	10.79 ± 1.65	0.799
BMI(kg/m^2^)	21.77 ± 3.31	26.59 ± 4.69	**< 0.001**
Family history of diabetes (n, %)	16.29%(50/307)	25.99%(46/177)	**< 0.001**
History of pregnancy disorders (n, %)	7.56%(27/357)	7.91%(14/177)	0.886
Parity (times)	1.20 ± 0.40	1.44 ± 0.50	**< 0.001**
*Ultrasound parameters*			
SAT(mm)	15.34 ± 5.32	23.19 ± 7.67	**< 0.001**
VAT(mm)	28.00 ± 9.81	41.05 ± 14.04	**< 0.001**

GDM, gestational diabetes mellitus; BMI, body mass index; SAT, subcutaneous adipose tissue; VAT, visceral adipose tissue.

Statistically, there were no significant differences in age, gestational weeks, and history of pregnancy disorders (*P* > 0.05). In the GDM group, the BMI (26.59 ± 4.69 vs. 21.77 ± 3.31 kg/m², *P* < 0.001), the incidence of family history of diabetes (25.99% vs. 16.29%, *P* < 0.001), and the parity (1.44 ± 0.05 vs. 1.20 ± 0.40, *P* < 0.001) were significantly higher.

When comparing the differences in ultrasonic parameters between the two groups, both the fetal SAT and VAT in the GDM group were higher than those in the control group. According to the independent samples *t* test, the differences were statistically significant (*P* < 0.001).

### Inverse probability weighting for bias correction

#### Propensity score and common support

To address the sampling bias from the higher-than-population GDM incidence in the cohort, Lasso logistic regression was used to calculate propensity scores. The optimal regularization parameter (λ_min = 0.022) was selected via 10-fold cross-validation. The propensity score distributions of the case (GDM) and control groups exhibited substantial overlap in the common support region (Supplementary Figure S1.1), satisfying the positivity assumption required for IPW.

#### Weight calculation and adjustment

Based on the propensity scores and assuming a target population GDM incidence of 10% (*P(GDM = 1)* = 0.1), initial sample weights were derived. The raw weights showed a long-tailed distribution with extreme values. After Winsorization (clipped at the 5th and 95th percentiles) and normalization, the weight distribution became more concentrated and reasonable (Supplementary Figure S1.2), ensuring stability in subsequent model training.

### Feature selection

Three feature selection methods were applied:

Genetic Algorithm: After five independent runs, the intersection of selected features consistently identified three core variables: BMI, SAT, and VAT (Supplementary Table S2.1 and Figure S2.1).

Recursive Feature Elimination : Cross-validation indicated that four features—SAT, VAT, age, and gestational weeks—yielded the highest AUC (Supplementary Figure S2.2).

Stepwise Regression: The minimal feature set selected by the AIC criterion comprised SAT and history of pregnancy disorders (Supplementary Table S2.2).

The GA-derived feature set (BMI, SAT, VAT) was chosen for final modeling because these features were consistently selected across all GA runs, are directly related to GDM pathophysiology, and provided a balance between predictive performance and model stability.

### Model performance on the internal test set

The default 0.5 threshold is often suboptimal for imbalanced data; therefore, the Youden index was used to determine the optimal cut-off for each model. Table 2 summarizes the performance of the 20 model combinations (five classifiers × four feature sets).

**Table 2:** Performance of machine-learning models on the internal test set.

Model	Youden’s Index	AUC	Accuracy	Balanced Accuracy	Sensitivity	Specificity	F1 Score	Macro F1	*P* (Delong’s test)*
XGBoost_GA	0.2734	0.9622	0.9244	0.921	0.90	0.942	0.9091	0.9222	Benchmark
XGBoost_FULL	0.3759	0.8849	0.8403	0.832	0.78	0.8841	0.8041	0.8347	<0.001
XGBoost_RFE	0.3620	0.8699	0.8403	0.8375	0.82	0.8551	0.8119	0.8366	<0.001
ANN_GA	0.0930	0.8609	0.8403	0.843	0.86	0.8261	0.8190	0.8381	<0.001
SVM_GA	0.3495	0.8310	0.7983	0.8041	0.84	0.7681	0.7778	0.7966	<0.001
SVM_FULL	0.3485	0.8246	0.7983	0.8068	0.86	0.7536	0.7818	0.7972	<0.001
MLR_GA	0.0970	0.8174	0.7899	0.7858	0.76	0.8116	0.7525	0.7850	<0.001
MLR_FULL	0.0875	0.8130	0.8067	0.8058	0.80	0.8116	0.7767	0.8032	<0.001
MLR_RFE	0.0790	0.8070	0.7983	0.8041	0.84	0.7681	0.7778	0.7966	<0.001
SVM_RFE	0.3947	0.8029	0.7311	0.7351	0.76	0.7101	0.7037	0.7288	<0.001
RF_GA	0.1271	0.8017	0.7395	0.7561	0.86	0.6522	0.7350	0.7394	<0.001
MLR_Step	0.0776	0.7946	0.7899	0.7968	0.84	0.7536	0.7706	0.7884	<0.001
ANN_Step	0.1246	0.7862	0.7815	0.7813	0.78	0.7826	0.7500	0.7780	<0.001
XGBoost_Step	0.0867	0.7806	0.7563	0.7623	0.80	0.7246	0.7339	0.7546	<0.001
ANN_FULL	0.0430	0.7758	0.7731	0.7823	0.84	0.7246	0.7568	0.7721	<0.001
RF_Step	0.1053	0.7725	0.7563	0.7678	0.84	0.6957	0.7434	0.7557	<0.001
RF_FULL	0.0551	0.7288	0.6639	0.6964	0.90	0.4928	0.6923	0.6610	<0.001
RF_RFE	0.1807	0.7099	0.7059	0.6913	0.60	0.7826	0.6316	0.6934	<0.001
ANN_RFE	0.1419	0.7001	0.6723	0.6816	0.74	0.6232	0.6549	0.6714	<0.001
SVM_Step	0.6270	0.4152	0.6218	0.5528	0.12	0.9855	0.2105	0.4810	<0.001

*Bonferroni-corrected *P*-values relative to XGBoost_GA.

Best-performing model: XGBoost with GA-selected features (XGBoost_GA) achieved the highest AUC (0.962), accuracy (0.924), balanced accuracy (0.921), sensitivity (0.90), and specificity (0.942).

Impact of feature selection: GA-based features consistently improved model performance; XGBoost_GA significantly outperformed both full-feature (XGBoost_FULL, AUC 0.885) and RFE-based models (XGBoost_RFE, AUC 0.870). Stepwise-selected features led to notably lower performance (e.g., XGBoost_Step AUC 0.781).

Classifier comparison: With the same GA features, XGBoost outperformed all other classifiers (ANN_GA AUC 0.861, SVM_GA AUC 0.831, MLR_GA AUC 0.817, RF_GA AUC 0.802).

Statistical significance: Delong tests with Bonferroni correction confirmed that XGBoost_GA’s AUC was significantly higher than that of every other model (all *P* < 0.05).

**Fig. 3:**
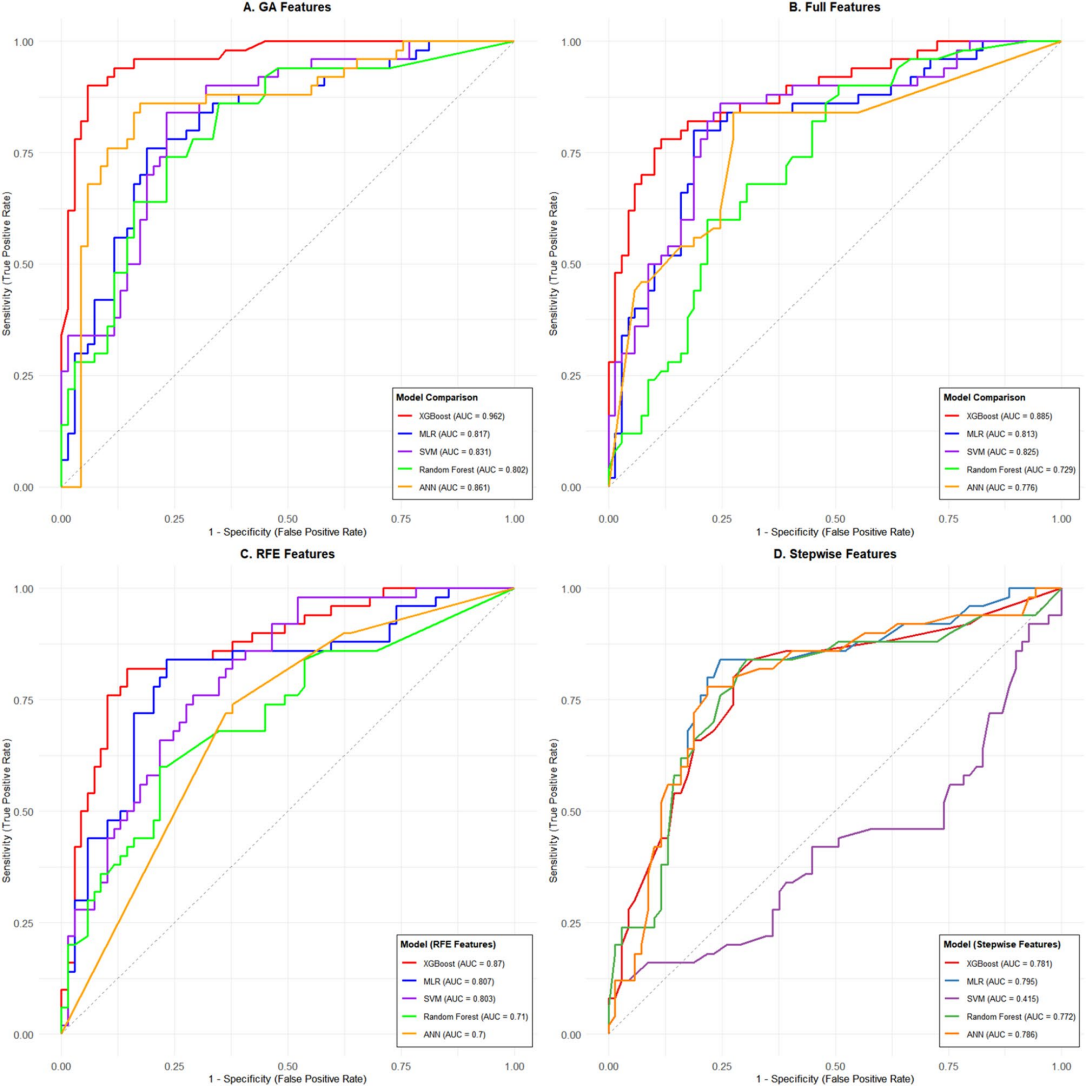
Comparison of ROC curves for different feature selection methods and machine‑learning models on the training set. The figure displays the receiver operating characteristic (ROC) curves for five machine‑learning models (XGBoost, MLR, SVM, Random Forest, ANN) across four feature selection strategies: (**A**) genetic algorithm (GA) selection; (**B**) all features (Full); (**C**) recursive feature elimination (RFE); and (D) stepwise regression (Stepwise). The area under the curve (AUC) for each model‑feature combination is annotated.

ROC curves for the four feature sets (Fig. 3) showed that models using GA-selected features (Panel A) clustered near the upper-left corner, with XGBoost_GA (AUC 0.962) being the top performer. In contrast, models built on stepwise-selected features (Panel D) displayed jagged curves and substantially lower AUCs, indicating instability due to excessive feature reduction.

**Fig. 4:**
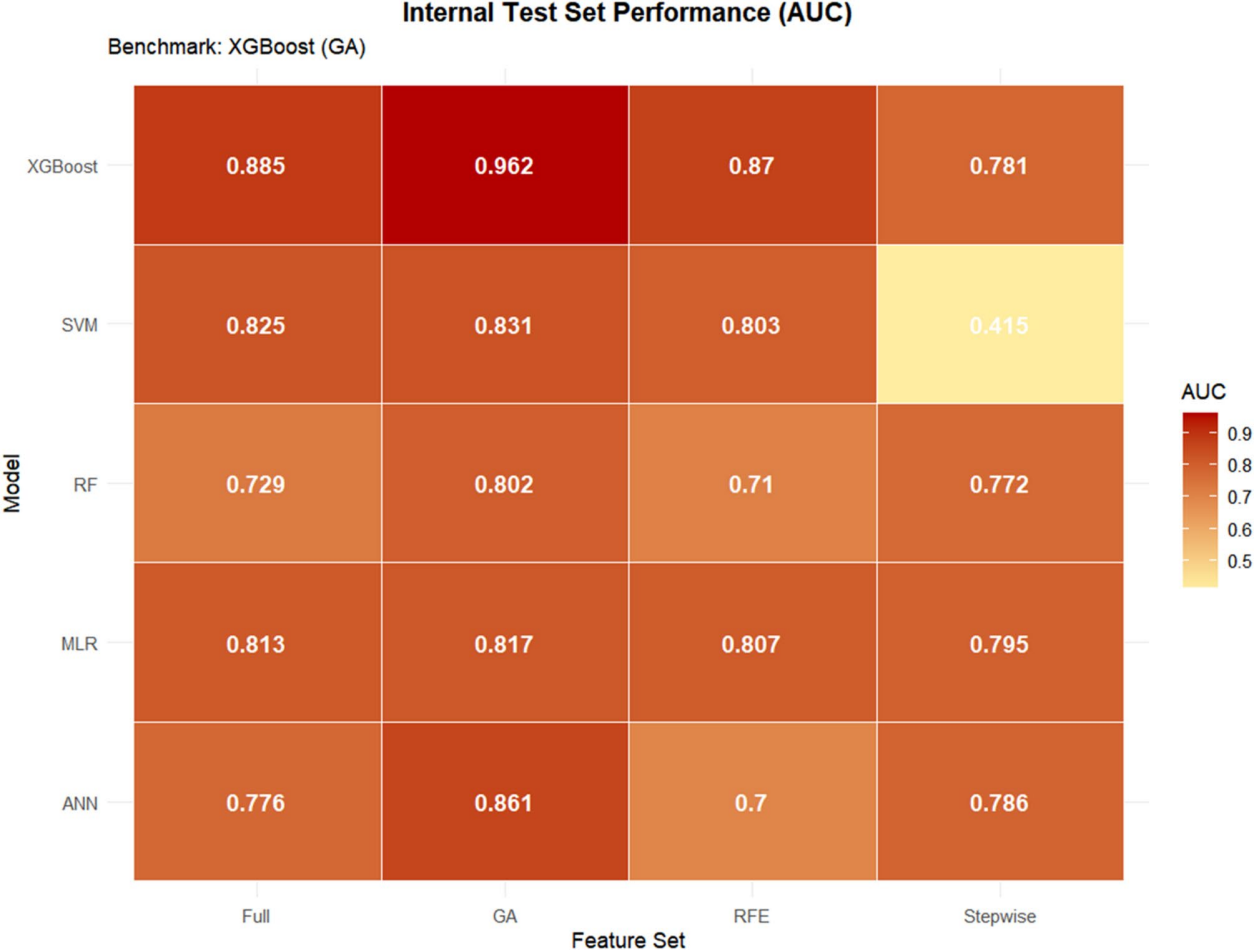
Heatmap of AUC performance for different feature selection methods and machine‑learning models on the internal test set. Color intensity corresponds to the AUC value, with darker red indicating higher performance. The XGBoost_GA combination shows the darkest shade (highest AUC), whereas SVM_Step shows the lightest shade (lowest AUC).

The corresponding heatmap (Fig. 4) visually reinforced these results: the GA column exhibited the deepest red hues (high AUCs), while the stepwise column was predominantly light-colored, especially for SVM_Step (AUC 0.415), highlighting the detrimental effect of over-reduction on support-vector machines.

### External validation

#### Overall performance

Models were further evaluated on an independent external cohort (Table 3). XGBoost_GA maintained robust predictive ability with an AUC of 0.878, accuracy of 0.885, sensitivity of 0.70, and specificity of 0.935. Although its AUC dropped slightly from the internal test set (0.962), the model showed no signs of severe overfitting and demonstrated good cross-dataset adaptability.

#### Robustness of feature selection methods

**Fig. 5:**
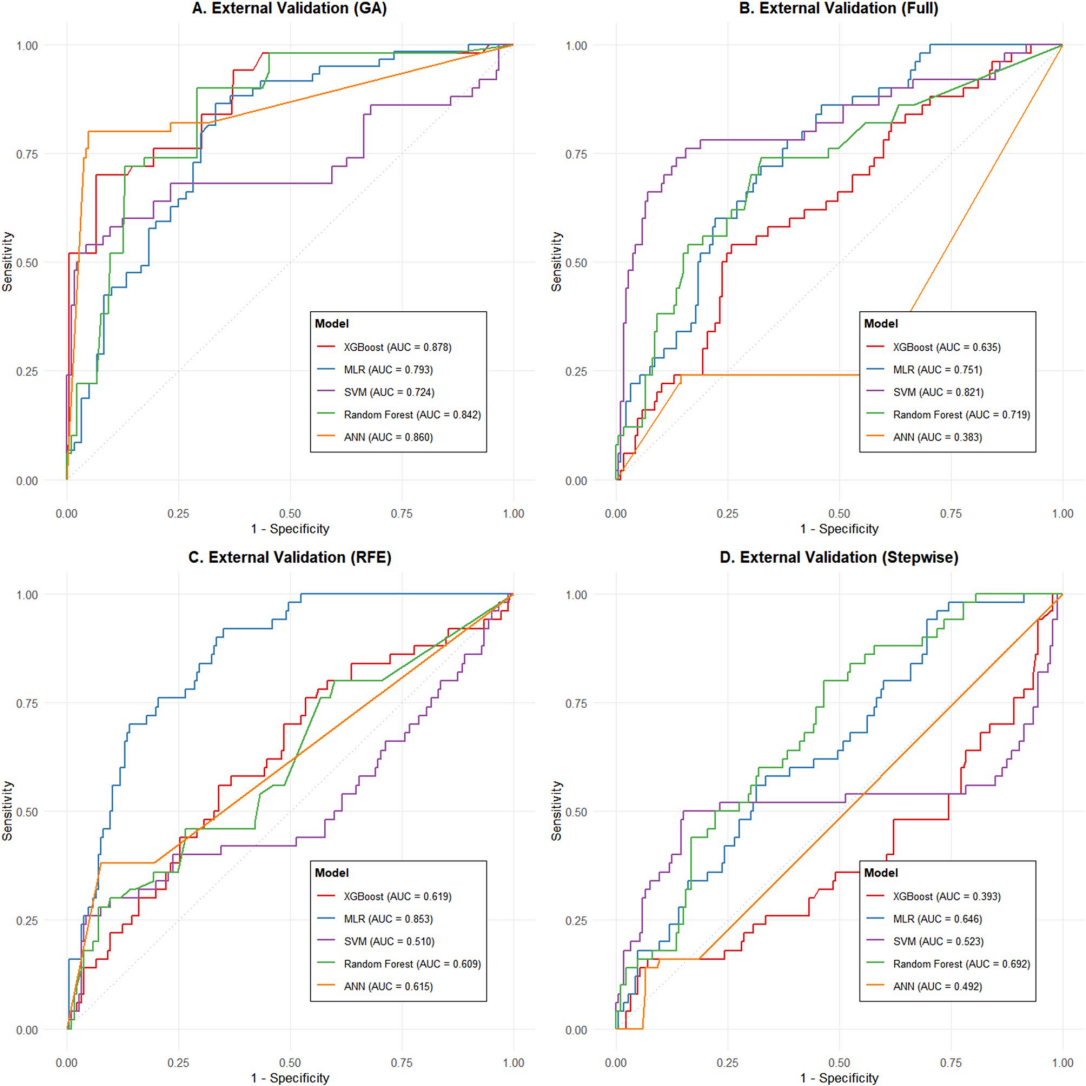
Comparison of ROC curves for different feature selection methods and machine‑learning models on the external validation set. ROC curves are shown for the same five machine‑learning models using the four feature selection approaches: (**A**) GA; (**B**) Full; (**C**) RFE; and (**D**) Stepwise. AUC values are indicated for each curve.

GA-based models (XGBoost_GA, ANN_GA, RF_GA) again showed smooth ROC curves near the upper-left corner (Fig. 5) with AUCs mostly above 0.8, confirming the stable predictive value of the core features (BMI, SAT, VAT) across populations. Conversely, models built on stepwise-selected features suffered a severe performance drop (e.g., XGBoost_Stepwise AUC 0.393, SVM_Stepwise AUC 0.523), indicating that oversimplified feature sets lack the generalizability needed for external data.

**Table 3:** Performance of machine-learning models on the external validation set.

Model	Threshold	AUC	Accuracy	Balanced Accuracy	Sensitivity	Specificity	F1 Score	Macro F1	*P* (Delong’s test)*
XGBoost_GA	0.3859	0.8782	0.8851	0.8176	0.70	0.9351	0.7216	0.8246	Bechmark
ANN_GA	0.9298	0.8603	0.9191	0.8757	0.80	0.9514	0.8081	0.8784	0.329
MLR_RFE	0.0990	0.8532	0.7064	0.7843	0.92	0.6486	0.5714	0.6741	0.234
RF_GA	0.0994	0.8423	0.7489	0.8041	0.90	0.7081	0.6040	0.7101	0.033
SVM_Full	0.3210	0.8214	0.8383	0.8024	0.74	0.8649	0.6607	0.7773	0.136
MLR_GA	0.0669	0.7934	0.7647	0.7655	0.8644	0.6667	0.7846	0.7627	<0.001
MLR_Full	0.0720	0.7510	0.6085	0.7003	0.86	0.5405	0.4831	0.5840	<0.001
SVM_GA	0.3183	0.7244	0.8681	0.7484	0.54	0.9568	0.6353	0.7774	<0.001
RF_Full	0.1167	0.7186	0.6894	0.7078	0.74	0.6757	0.5034	0.6387	<0.001
RF_Stepwise	0.0871	0.6920	0.5915	0.6676	0.80	0.5351	0.4545	0.5640	<0.001
MLR_Stepwise	0.1124	0.6457	0.6596	0.6232	0.56	0.6865	0.4118	0.5861	<0.001
XGBoost_Full	0.0532	0.6348	0.6979	0.6403	0.54	0.7405	0.4320	0.6131	<0.001
XGBoost_RFE	0.0089	0.6194	0.5277	0.6124	0.76	0.4649	0.4064	0.5071	<0.001
ANN_RFE	0.5262	0.6153	0.8085	0.6522	0.38	0.9243	0.4578	0.6708	<0.001
RF_RFE	0.2230	0.6089	0.7915	0.6049	0.28	0.9297	0.3636	0.6195	<0.001
SVM_Stepwise	0.3402	0.5235	0.7745	0.6743	0.50	0.8486	0.4854	0.6705	<0.001
SVM_RFE	0.4026	0.5101	0.8085	0.6084	0.26	0.9568	0.3662	0.6267	<0.001
ANN_Stepwise	0.2311	0.4921	0.7660	0.5376	0.14	0.9351	0.2029	0.5329	<0.001
XGBoost_Stepwise	0.5382	0.3930	0.7660	0.5449	0.16	0.9297	0.2254	0.5438	<0.001
ANN_Full	0.1608	0.3831	0.7234	0.5470	0.24	0.8541	0.2697	0.5495	<0.001

*Compared with XGBoost_GA.

**Fig. 6:**
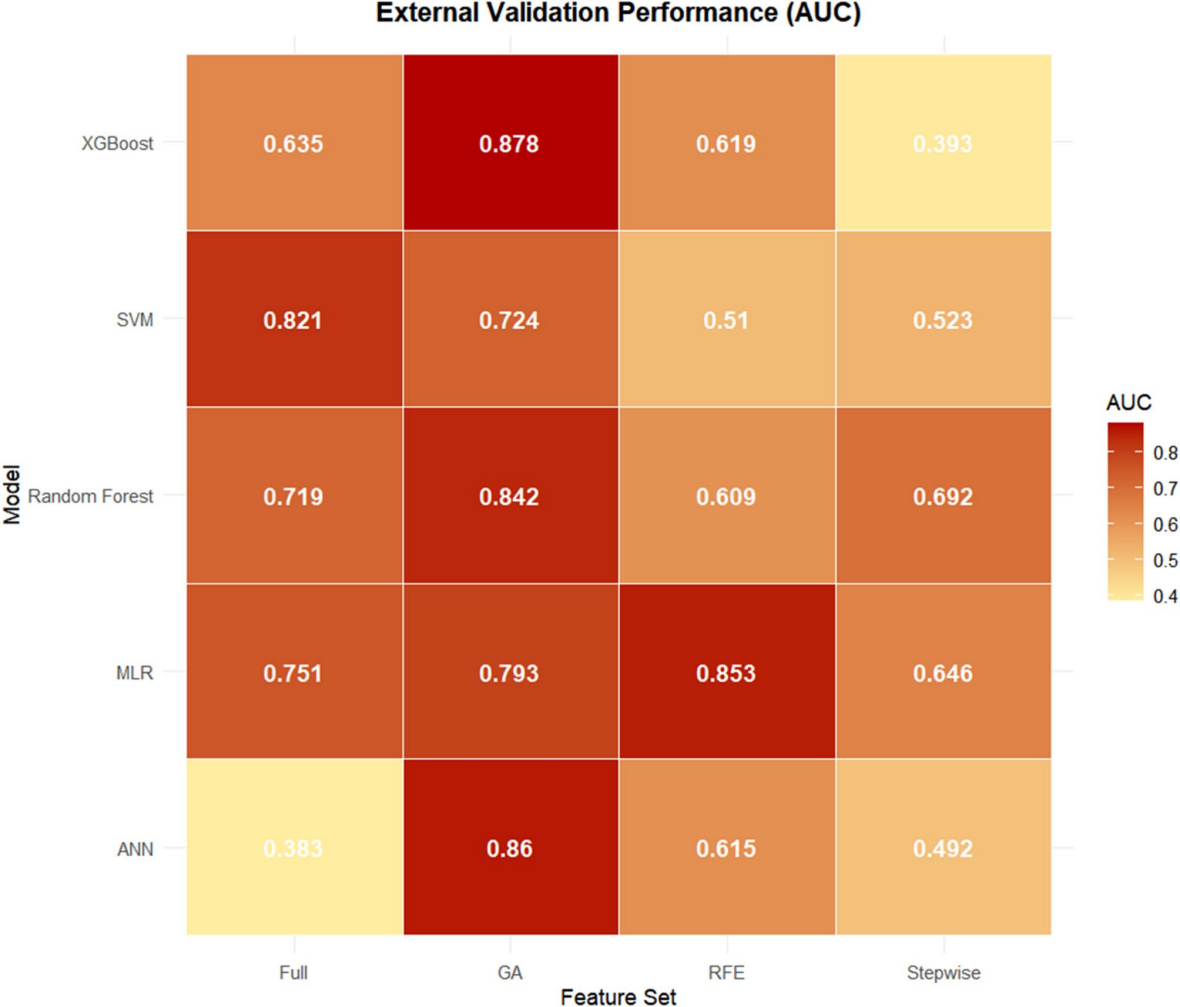
Heatmap of AUC performance for different feature selection methods and machine‑learning models on the external validation set. The color gradient reflects AUC values, with deep red representing the highest performance. XGBoost_GA occupies the darkest cell (AUC = 0.878), while ANN_Full corresponds to the lightest cell (AUC = 0.383).

The external-validation heatmap (Fig. 6) further illustrated that the GA column contained the highest AUCs, while the stepwise column was predominantly pale, underscoring the superior generalizability of GA-selected features.

#### Statistical significance in external validation

Delong tests on the external set revealed that ANN_GA (*P* = 0.329), MLR_RFE (*P* = 0.234), and SVM_Full (*P* = 0.136) did not differ significantly from the benchmark XGBoost_GA. In contrast, all stepwise-based models and the full-feature XGBoost_Full performed significantly worse (all *P* < 0.001), reinforcing the necessity of GA feature selection within the XGBoost framework.

### Robustness testing

A series of sensitivity analyses confirmed the robustness of the proposed model. The performance of XGBoost_GA remained optimal with minimal fluctuation when the random seed was altered and the training-to-test ratio was adjusted to 0.7/0.3. Modifying the prior probability in inverse probability weighting (*P* = 0.04 or 0.07) had a negligible impact on model performance. Furthermore, when a weighted ensemble approach was applied, the ensemble built on GA-selected features significantly outperformed those based on other feature sets in both internal and external validation (all *P* < 0.05). Together, these results demonstrate that the model maintains stable performance across varying data splits, weighting assumptions, and ensemble strategies.

## Discussion

This study systematically evaluated the predictive value of early-pregnancy clinical and ultrasonographic indicators for GDM. By integrating genetic algorithm based feature selection with multiple machine learning models, we successfully developed and validated a robust GDM risk prediction model. BMI, VAT, and SAT were identified as highly stable core predictors. The model demonstrated exceptional discriminative ability in the internal test set and maintained good generalizability in an independent external validation set, providing strong technical support for opportunistic screening of GDM using routine first-trimester data in clinical practice, particularly in resource-limited settings.

Our study contributes to the development of early GDM prediction in Chinese populations. While existing models mainly use clinical and laboratory data^13–15^, our model incorporates first-trimester ultrasound measurements of visceral and subcutaneous adipose tissue thickness. This approach utilizes the concept of opportunistic screening by repurposing routinely acquired imaging data for risk assessment. Compared to traditional logistic regression models, our machine learning approach (XGBoost) could potentially capture more complex relationships among variables.

As a well-established indicator of adiposity, BMI retained significant value in predicting GDM, with mechanisms involving chronic inflammation, mitochondrial dysfunction, and adipose tissue hypoxia^16–18^. Notably, several multi-ethnic studies, particularly those conducted in Western populations, reported a relatively lower population attributable risk (PAF) of BMI for GDM among Asian women^19^. This apparent discrepancy was explained by the observation that Asian women developed GDM at substantially lower BMI thresholds than other ethnic groups. Rather than contradicting the value of BMI, this finding shifted the etiological emphasis from overall adiposity to fat distribution patterns—a perspective supported by growing evidence^20,21^. In line with this, our study demonstrated that visceral adipose tissue thickness also possessed significant predictive ability.

Visceral fat accumulation can trigger a series of metabolic disorders. Excessive visceral fat prompts adipocytes to secrete inflammatory factors, such as tumor necrosis factor-α and interleukin-6. These inflammatory factors interfere with the normal insulin signaling pathway, reducing cellular sensitivity to insulin and leading to insulin resistance. Insulin resistance is a critical factor in the development of gestational diabetes mellitus. To maintain normal blood glucose levels, the body compensatorily increases insulin secretion. When the pancreas cannot meet this demand, blood glucose levels rise, ultimately resulting in GDM. Multiple prospective cohort studies have confirmed a significant positive correlation between early-pregnancy visceral fat thickness and the subsequent risk of GDM, consistent with the findings of this study^22–24^. Compared to traditional waist circumference measurements, the quantitative assessment of visceral fat thickness more accurately reflects metabolic risk, which explains its strong predictive ability in the prediction model.

Subcutaneous fat thickness is a significant indicator for predicting gestational diabetes mellitus, which aligns with the findings of Nassr et al. ^25^. Their study demonstrated through ultrasound measurements that the predictive efficacy of subcutaneous fat for GDM is markedly superior to that of the traditional BMI indicator^25^. Mechanistically, excessive proliferation of subcutaneous adipose tissue, functioning as an active endocrine organ, leads to disordered secretion of adipokines. This includes a reduction in adiponectin levels and the development of leptin resistance, while simultaneously promoting the release of pro-inflammatory factors such as IL-6 and MCP-1, which collectively exacerbate the insulin-resistant state during pregnancy. In contrast to previous studies that primarily focused on overall obesity indicators, our results further affirm the unique value of fat distribution characteristics in predicting GDM. Subcutaneous fat indicators effectively address the limitations of BMI in distinguishing fat distribution and provide a more accurate reflection of variations in fat distribution.

It should be noted that the novelty of this study lay not in identifying new risk factors, but in developing a prediction model for the Northern Chinese pregnant population that combined routinely available ultrasound-derived abdominal fat distribution indicators (VAT/SAT) with BMI, tailored for first-trimester opportunistic screening. In regions with relatively limited clinical resources, such a model based on the most basic and accessible data holds practical value, enabling early risk stratification without increasing the medical burden.

While this pragmatic approach defines the core utility of our model, a closer examination of its predictive performance reveals important boundaries. Analysis of model errors clarifies its clinical scope. False positives may occur in women with high adiposity but preserved metabolic health, or in cases where early lifestyle intervention prevented GDM. False negatives may reflect GDM driven by non-adiposity factors (e.g., genetics) or metabolic changes not yet evident in early fat distribution. Therefore, this model is best used for initial risk stratification rather than definitive diagnosis.

Several limitations should be noted. First, the predictive feature set was constrained by both practical considerations for resource-limited settings and data availability, particularly the limited early-pregnancy metabolic markers. Additionally, as the model was developed in a Northern Chinese cohort, its application to other populations requires external validation and potential recalibration. Future studies should incorporate more comprehensive metabolic and genetic indicators where feasible and establish standardized imaging protocols across centers.

## Conclusion

This study integrated genetic algorithm-based feature selection with multiple machine-learning models and leveraged routinely available first-trimester clinical and ultrasonographic indicators to successfully develop and validate an early GDM prediction model in Northern Chinese pregnant women. The model demonstrated excellent discriminatory performance and high generalization stability, providing an effective tool for implementing low‑cost, non‑invasive opportunistic screening in clinical practice.

## Supplementary Information

Below is the link to the electronic supplementary material.

Supplementary Material 1

## Data Availability

The de-identified datasets generated and analyzed during this study are available from the corresponding author upon reasonable request, subject to institutional review board approval and data sharing agreements.
