# Supplementary material for: Opportunistic screening data for early prediction of GDM in Northern Chinese women: a multicenter machine learning study
Source: Sci Rep. 2026 Mar 9;16:12818. doi: 10.1038/s41598-026-42700-y (PMC13096644; doi:10.1038/s41598-026-42700-y)
Supplement: Supplementary file 1 — Supplementary Material 1 [file 41598_2026_42700_MOESM1_ESM.docx]

**Supplementary Material**

## **Supplementary Material 1: IPW Bias Adjustment and Data Preprocessing**


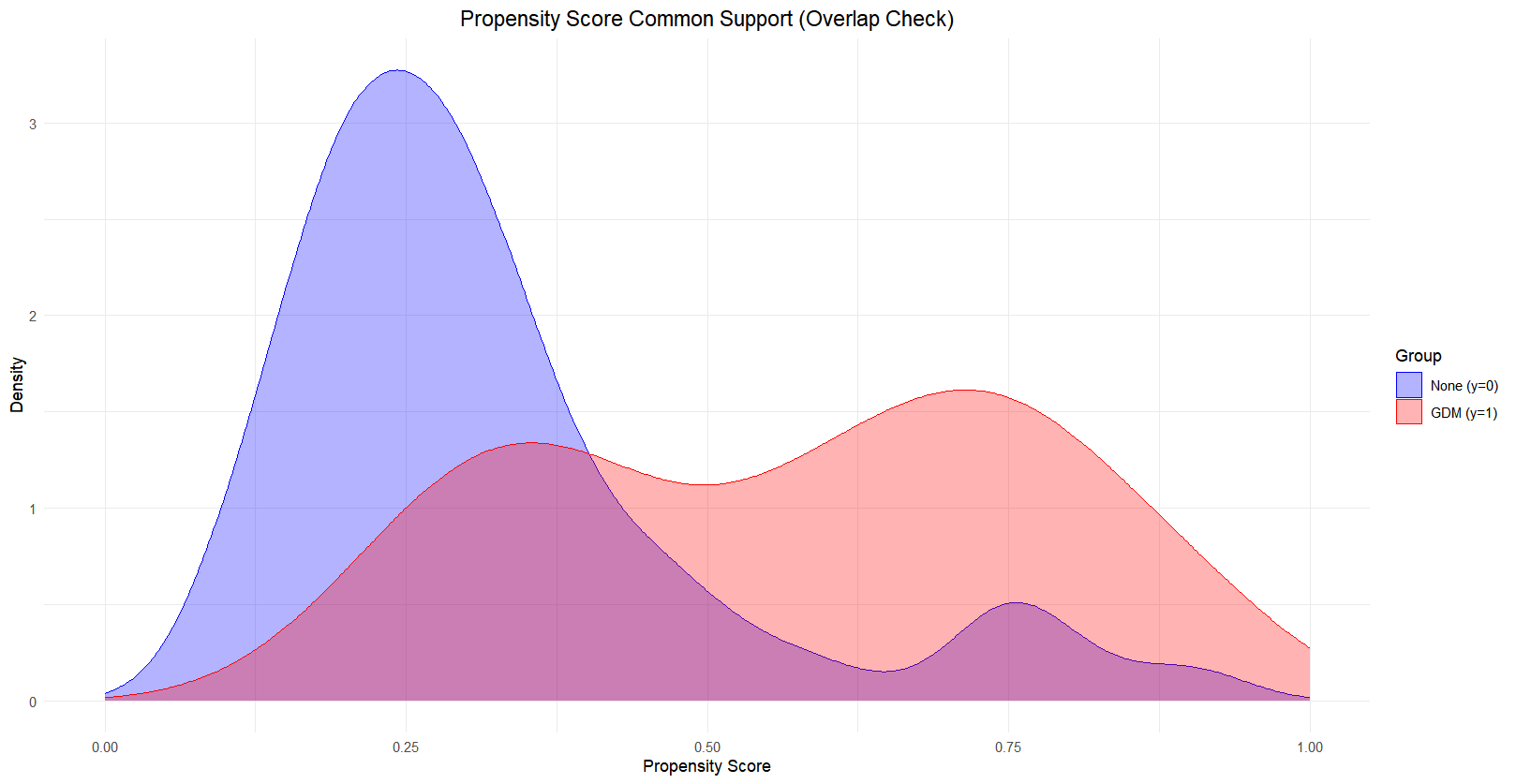


Figure S1.1 Distribution of propensity scores before and after IPW adjustment.
Density plots showing the propensity score distributions for the GDM group (red) and the control group (blue) before (A) and after (B) Inverse Probability Weighting (IPW). The overlap region satisfies the common support assumption, confirming the validity of IPW for bias adjustment.


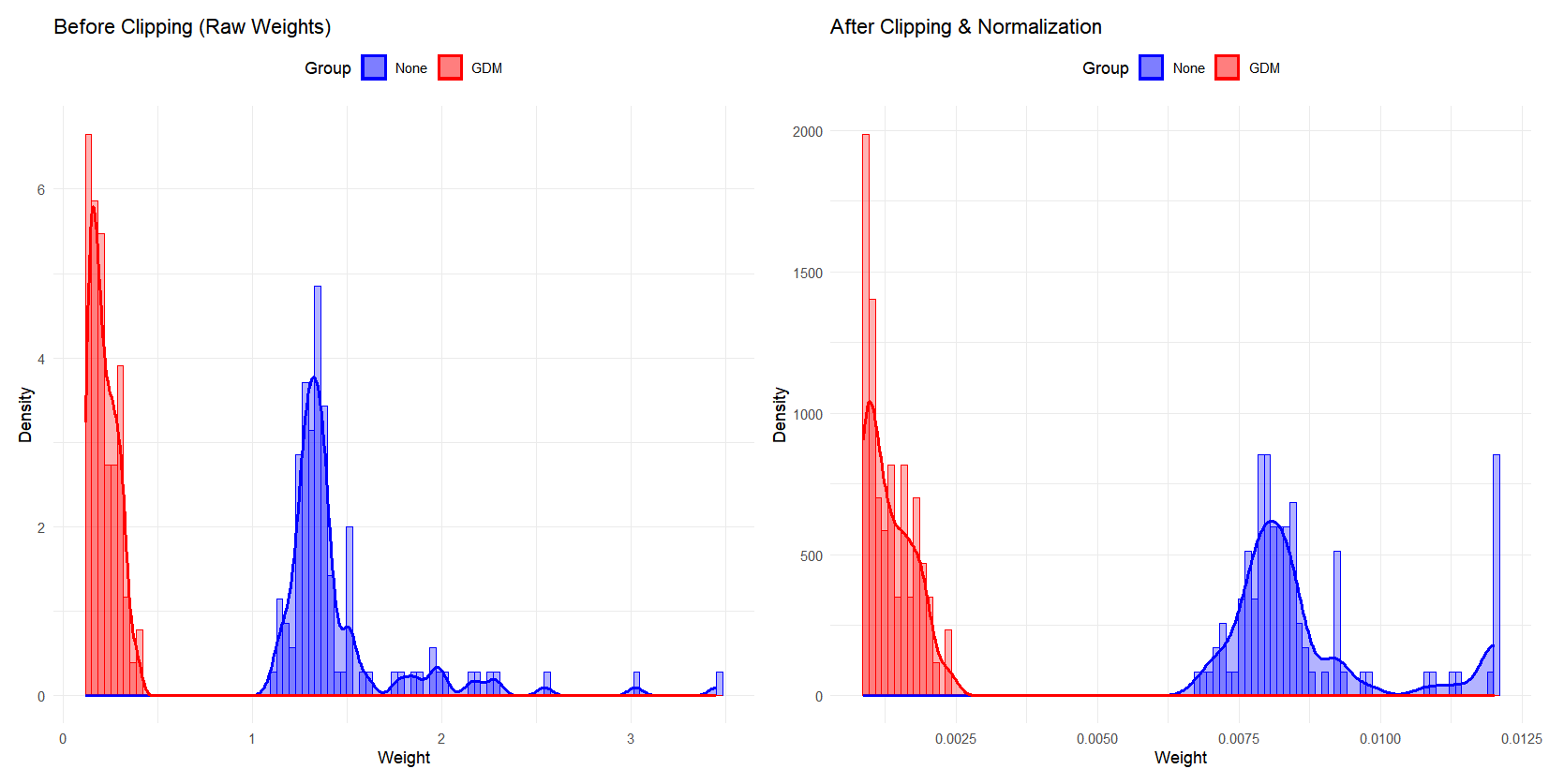


Figure S1.2 Distribution of IPW weights before and after winsorization.

Left panel shows the original weight distribution (long-tailed), right panel shows the distribution after winsorization (5th–95th percentiles) and normalization. Winsorization stabilizes model training by reducing extreme weight values.

**Supplementary Material 2: Feature Selection Process and Algorithm Details**


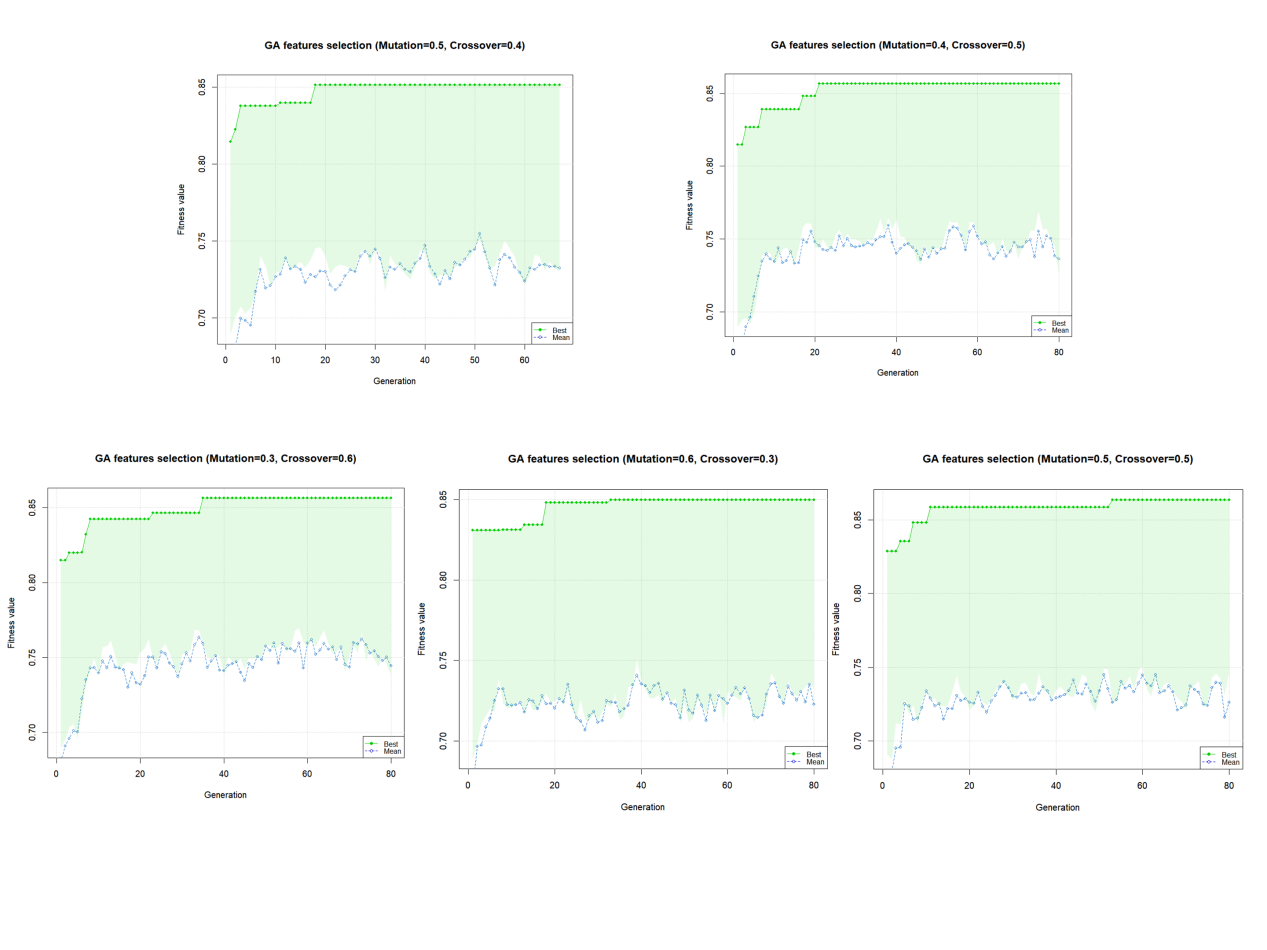


Figure S2.1. Convergence plots of Genetic Algorithm (GA) feature selection under five different parameter combinations.

Each subplot shows the evolution of the best fitness value (AUC, left Y-axis) and the number of selected features (right Y-axis) across generations for a specific combination of mutation and crossover rates. The algorithm stabilizes around the 10th–20th generation, with consistent selection of BMI, VAT, and SAT.


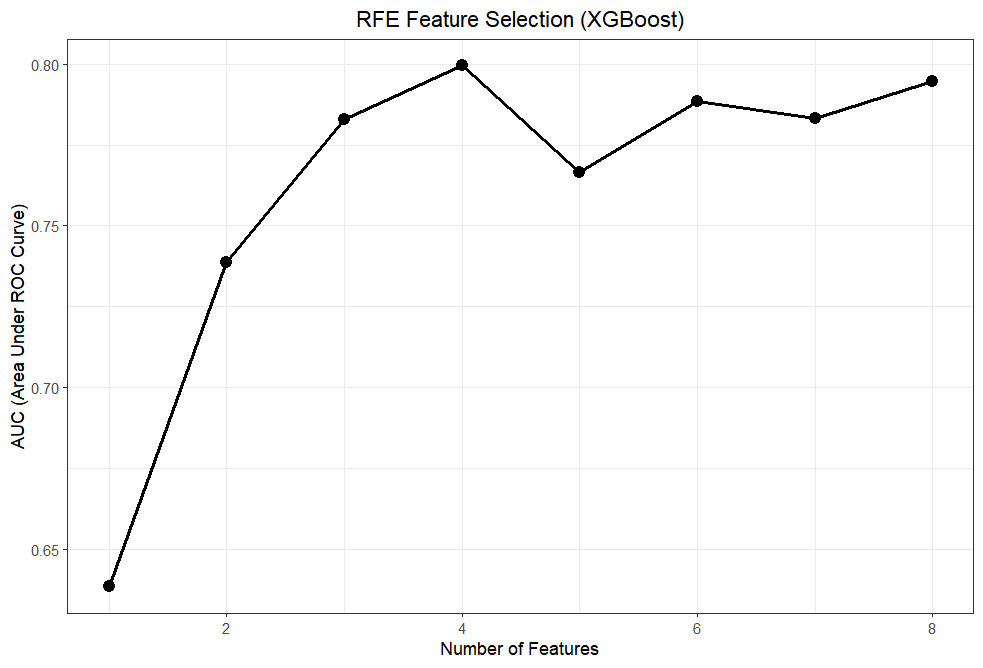


Figure S2.2. Recursive Feature Elimination (RFE) selection curve.

Relationship between the number of features and cross-validated AUC. The optimal feature count is 4 (SAT, VAT, Age, Gestational Weeks), beyond which additional features reduce model performance, indicating overfitting.

Table S2.1. GA feature selection results under different crossover probabilities.

| Combination | Selected Features |
| --- | --- |
| Mutation=0.5, Crossover= 0.4 | SAT, VAT, BMI |
| Mutation=0.4, Crossover= 0.5 | SAT, VAT, BMI, History of Pregnancy Disorders |
| Mutation=0.3, Crossover= 0.6 | SAT, VAT, BMI, Diabetes |
| Mutation=0.6, Crossover= 0.3 | Gestational Week, SAT, VAT, BMI |
| Mutation=0.5, Crossover= 0.5 | SAT, VAT, BMI |
|  | SAT, VAT, BMI |

Table S2.2. Detailed results of RFE and Stepwise Regression feature selection.

| Method | Selected Features | Performance Metric | Number of Features |
| --- | --- | --- | --- |
| RFE (XGBoost) | SAT, VAT, Age, Gestational Weeks | AUC = 0.87 | 4 |
| Stepwise Regression | SAT, History of Pregnancy Disorders | AIC = 29.50 | 2 |
